# A Novel Two-Stage Heat Treatment with Medium-Temperature Aging Influence on Microstructure, Al_3_(Sc, Zr) Nanoprecipitation, and Application Properties, Enhancing Selective Laser Melting of Al–Mg–Sc–Zr Alloy

**DOI:** 10.3390/nano12122078

**Published:** 2022-06-16

**Authors:** Jun-Ren Zhao, Liang-Yan Lee, Kai-Chieh Chang, Fei-Yi Hung

**Affiliations:** Department of Materials Science and Engineering, National Cheng Kung University, Tainan 701, Taiwan; a2x346yz03@gmail.com (J.-R.Z.); gogogo2315@gmail.com (L.-Y.L.); eran871003@gmail.com (K.-C.C.)

**Keywords:** Al–Mg–Sc–Zr, selective laser melting (SLM), heat treatment, medium-temperature aging, high-temperature tensile, rotation fatigue

## Abstract

Al–Mg–Sc–Zr alloy fabricated through selective laser melting (SLM) is an additive manufacturing alloy with promising industrial potential. In this study, as-printed specimens were subjected to either single-stage or two-stage heat treatment processes to investigate the effect of temperature from room temperature to high temperature on the specimens’ tensile and fatigue properties to establish a reliable reference for aerospace applications. The tensile test results indicated that the heat treatment contributed to determine the properties of the nanoprecipitate Al_3_(Sc, Zr) with a strengthening phase, improving tensile strength. Moreover, the dynamics strain aging (DSA) effect vanished as temperature increased. It is noteworthy that the nanoprecipitation was distributed at the boundary of the melting pool after single-stage heat treatment with the highest tensile properties in all tests. In addition, the microstructure observed after the two-stage heat treatment indicated a melting pool interface decomposition, and the nanoprecipitation was homogeneously scattered over the Al matrix, increasing strength and further delaying fatigue crack transmission. Those features build a high-fatigue-resistance foundation. TEM analysis also confirmed the promotion of Sc thermal diffusion and an Al_3_(Sc, Zr) precipitation transformation mechanism under two-stage heat treatment, corresponding to aforementioned inferences. The SLM Al–Mg–Sc–Zr alloy with two-stage heat treatment brings about balance between tensile properties and fatigue resistance, providing new insight into additive manufacturing with Al alloys.

## 1. Introduction

Considering high corrosion-resistance and ductility, Al–Mg alloys have been widely utilized in the automobile, shipbuilding, and aerospace industries [1,2,3,4,5]. According to the literature [6,7,8,9], combining Sc and Zr elements with Al–Mg alloys to form Al–Mg–Sc–Zr alloys can precipitate Al_3_(Sc, Zr) after heat treatment, thereby improving mechanical properties. Previous study has revealed that the Al_3_(Sc, Zr) precipitation’s crystal structure is similar to α-Al, which can increase matrix strength and reduce grain coarseness [10]. These characteristics set Al–Mg–Sc–Zr alloys as high-strength application potential material.

To date, many researchers have mentioned the heat treatment and the mechanical properties of SLM Al–Mg–Sc–Zr alloys [2,3,8,11,12], and the single-stage heat treatment process SLM Al–Mg–Sc–Zr alloy is subjected to causes it to gain strength but lose ductility [12]. It is a remarkable fact that the single-stage heat treatment process neither homogenizes SLM Al–Mg–Sc–Zr alloys, nor does the residual melting pool interface resist crack propagation effectively. In view of these disadvantages in the single-stage heat treatment process, this study introduces heat treatment comprised of solution treatment and medium-temperature aging, which form a two-stage heat treatment process, achieving homogeneous and high-fatigue-resistance materials. 

SLM technology has widely been introduced to fabricate various aluminum alloys, such as Al–Si, Al–Mg, and Al–Zn alloys [13,14,15,16,17]; however, these alloys are not characterized by high-temperature applications [18,19]. In terms of high-temperature applications, the literature has pointed out the traditional extrusion Al–Mg–Sc alloys with excellent superplasticity in the range of 250-500 °C [20]. Even now, investigation through heat treatment and high-temperature mechanical properties of SLM Al–Mg–Sc–Zr alloys is still lacking; in addition, the interaction of Al_3_(Sc, Zr) precipitates and the melting pool structure, which affects fatigue resistance, demands further research [9,21,22]. Taking the above reasons into account, systematically investigating high-temperature properties and evaluating their fatigue characteristics are essential. This study compares the microstructure and precipitation of SLM Al–Mg–Sc–Zr alloy as-printed specimens, single-stage-heat-treated specimens, and two-stage-heat-treated specimens to evaluate the mechanical properties at room and high temperatures (RT-350 °C) as well as clarify fatigue life and failure mechanism. Notably, TEM analysis is applied to confirm the phase transformation mechanism resulting from two-stage heat treatment of the SLM Al–Mg–Sc–Zr alloy. The relevant results include significant discoveries, providing reference for the application of SLM Al–Mg–Sc–Zr alloy in the automobile, shipbuilding, and aerospace industries.

## 2. Experimental Procedure

**Figure 1:**
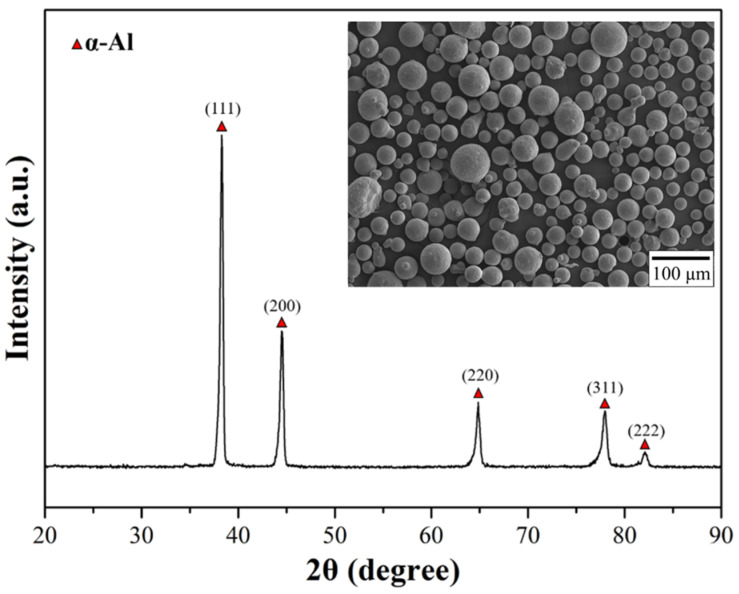
SEM and XRD analysis of the Al–Mg–Sc–Zr powders.

**Figure 2:**
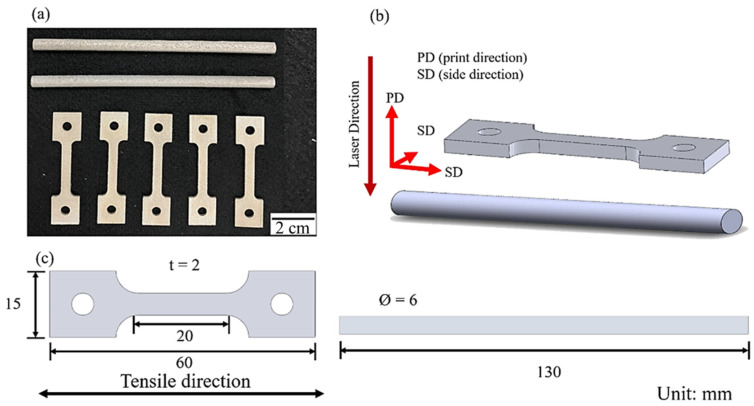
Schematic of the specimen of the SLM Al–Mg–Sc–Zr alloy: (**a**) macroscopic morphology, (**b**) manufacturing direction, and (**c**) size of tensile specimen and fatigue specimen.

The SLM Al–Mg–Sc–Zr alloys in this study are fabricated by ANJI Technology Co., Ltd (Tainan, Taiwan). The printing parameters and chemical composition are listed in Table 1 and Table 2, respectively [16,21]. The average size of the prealloyed powder is 30 μm (Figure 1). According to the results of the X-ray diffraction (XRD) used to analyze the phase composition, the powder has no clear precipitation phase [23]. The appearance, printing direction, and the dimensions of the tensile and fatigue specimens are shown in Figure 2a–c. 

In this study, as-printed referred to SLM Al–Mg–Sc–Zr alloy before heat treatment. The as-printed specimens were subjected to single-stage heat treatment at 350 °C for 6 h [3,6,18] and the two-stage heat treatment: solid solution heat treatment at 500 °C for 1 h and aging treatment at 350 °C for 6 h [24,25,26]. The heat treatment parameters are listed in Table 3.

These specimens were ground through #80 to #4000 SiC sandpaper in sequence and polished using 1 μm, 0.3 μm Al_2_O_3_, and 0.04 μm SiO_2_ to polish. Finally, etching was performed with a solution of 5 mL of HNO_3_ + 3 mL of HCl + 2 mL of HF + 190 mL of H_2_O. Optical microscope (OM, OLYMPUS BX41M-LED, Tokyo, Japan) and XRD spectroscopy (Bruker AXS GmbH, Karlsruhe, Germany) were employed to analyze the phase composition.

HRF hardness measurement was conducted by using a hardness machine (Mitutoyo AR-10, Kanagawa, Japan). Universal testing machine (HUNGTA, HT-8336, Taichung, Taiwan) was used for tensile testing the strength with a strain rate of 1 mm/min and an initial strain rate of 1.83 × 10^−3^ s^−1^ at both room temperature and high temperatures in the range of 100–350 °C [20].

A rotating fatigue-testing machine (HUNGTA HT-810, Taichung, Taiwan) with loadings of 7, 12, 17, and 22 kg (i.e., stress of 33.01, 56.59, 80.17, and 103.75 kg/mm^2^) established fatigue properties [21]. In addition, fatigue resistance was compared using a Stress–Number of cycles to failure curve (S-N curve). A scanning electron microscope (HITACHI SU-5000, HITACHI, Tokyo, Japan) inspected the fatigue fracture surface and explored the fracture mechanism. An electron probe microanalyzer (EPMA, JEOL JXA-8900R, Taipei, Taiwan) was used to compare the distribution of the alloying elements between as-printed and two-stage-heat-treated specimens. Finally, a transmission electron microscope (JEM-2010-200 KV, JEOL Ltd., Tokyo, Japan) with an energy dispersive spectrometer (EDS) was further applied to clarify the thermal diffusion mechanism of elements in the two-stage heat treatment process.

## 3. Results and Discussion

### 3.1. Microstructure and Phase Analysis

**Figure 3:**
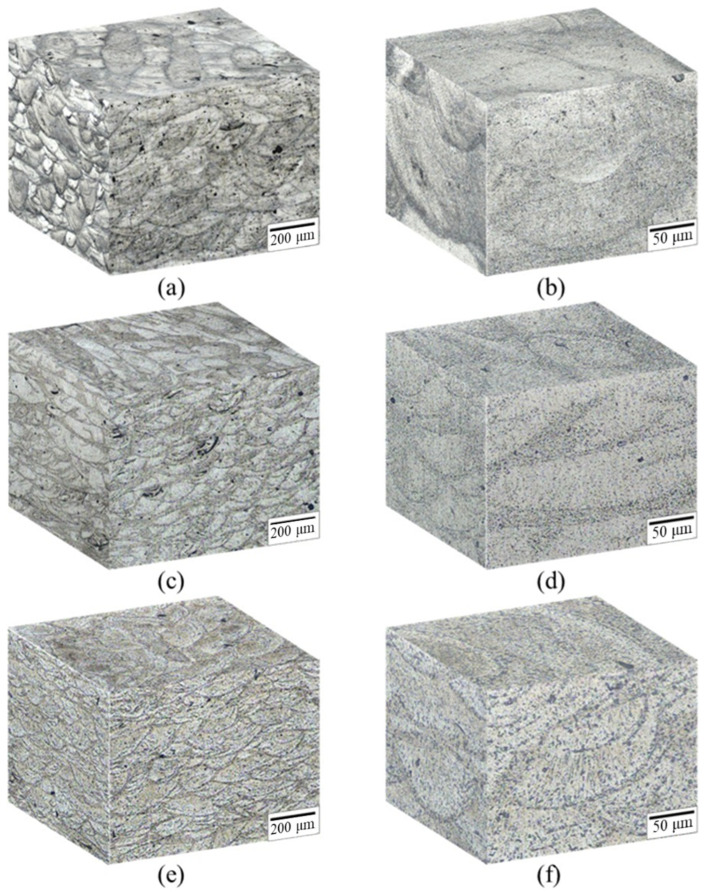
Three-dimensional microstructure of the SLM Al–Mg–Sc–Zr alloys: (**a**,**b**) as-printed, (**c**,**d**) single-stage heat-treated, (**e**,**f**) two-stage heat-treated specimens.

Figure 3a,b displays the microstructure of as-printed SLM Al–Mg–Sc–Zr alloy, which exhibited typical melting pool structure similar to that of other SLM Al alloys [13,16,21]. The width and depth of the melting pool were approximately 200 and 100 μm, respectively. Figure 3c,d presents the microstructure of the single-stage-heat-treated specimens with obvious melting pool structure the same as what the as-printed specimens showed, which indicated that the process could not completely decompose the melting pool structure. Figure 3e,f shows the microstructure of the specimens subjected to two-stage heat treatment. Observed by 3D metallographic diagram, the melting pool structure partially decomposed after high-temperature solid solution treatment was confirmed.

**Figure 4:**
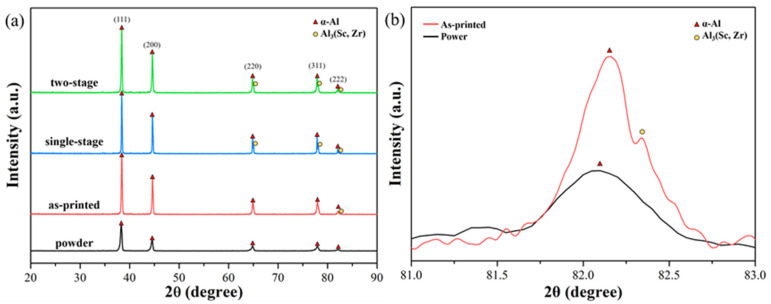
(**a**) X-ray diffraction pattern of the SLM Al–Mg–Sc–Zr alloys, obtained over a wide range of 2θ values. (**b**) X-ray diffraction pattern in the vicinity of the peak of α-Al (2θ = 82°).

XRD results are shown in Figure 4a for all specimens. Compared with the powder XRD results (Figure 1), double diffraction peaks corresponding to the (311) and (222) crystal planes were observed (Figure 4b). According to the literature [9,27], the double peaks represent precipitation of Al_3_(Sc, Zr) resulting from residual heat throughout the SLM process, and the peaks of Al_3_(Sc, Zr) were clearly divided after heat treatments. This indicates that Al_3_(Sc, Zr), serving as a strengthening phase, precipitated either in single-stage or in two-stage heat treatment to improve the mechanical properties of alloys [9].

### 3.2. Mechanical Properties at Room Temperature

**Figure 5:**
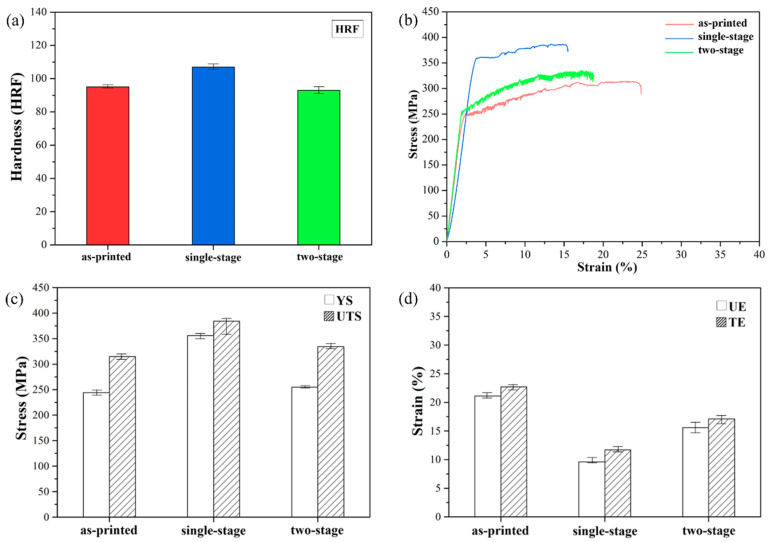
(**a**) Hardness, (**b**) tensile stress–strain curve, (**c**) tensile strength, and (**d**) elongation of the SLM Al–Mg–Sc–Zr alloys at room temperature (YS: yield strength, UTS: ultimate tensile strength, UE: uniform elongation, and TE: total elongation).

Figure 5a compares the hardness (HRF) results of all specimens. After the single-stage heat treatment, Al_3_(Sc, Zr) precipitated at the melting pool boundary, increasing the hardness of SLM Al–Mg–Sc–Zr alloys from HRF95 to HRF107. On the other hand, the melting pool was decomposed and the Sc and Zr elements were dissolved into the α-Al matrix after the two-stage heat treatment. Simultaneously, a precipitation process involving Al_3_(Sc, Zr)-strengthening precipitation occurred, producing strength and bringing about the observed slight change in hardness.

Figure 5b presents the tensile curves of the SLM Al–Mg–Sc–Zr alloys. All specimens exhibited jagged characteristics of dynamic strain aging (DSA) at room temperature [6,11]. The principle of the DSA phenomenon was the dissolved Mg in Al–Mg–Sc–Zr alloys forming a dislocation atmosphere, causing the stress and strain to be released in stages [28,29]. After solid solution treatment, more Mg element dissolved into the α-Al matrix, promoting the more significant jitter phenomenon and ductility improvement (Figure 5c,d, data lists in Table 4) [29,30,31]. The single-stage-heat-treated specimens revealed the highest strength but the lowest ductility due to the presence of grain boundaries, of melting pool boundaries, and of Al_3_(Sc, Zr) precipitation under medium-temperature aging treatment. Therefore, the strength increased, but the ductility could not meet standards of industrial application, and the tensile strength was limited in the printing direction [9,21]. Notably, the two-stage heat treatment process with solid solution effect contributes to the decomposition of melting pool boundaries, alloying element homogeneous dissolution, and reduction in the dependence of the tensile failure direction [25,26]. Thus, the two-stage-heat-treated specimens exhibited a better combination of tensile strength and ductility.

### 3.3. High-Temperature Mechanical Properties

**Figure 6:**
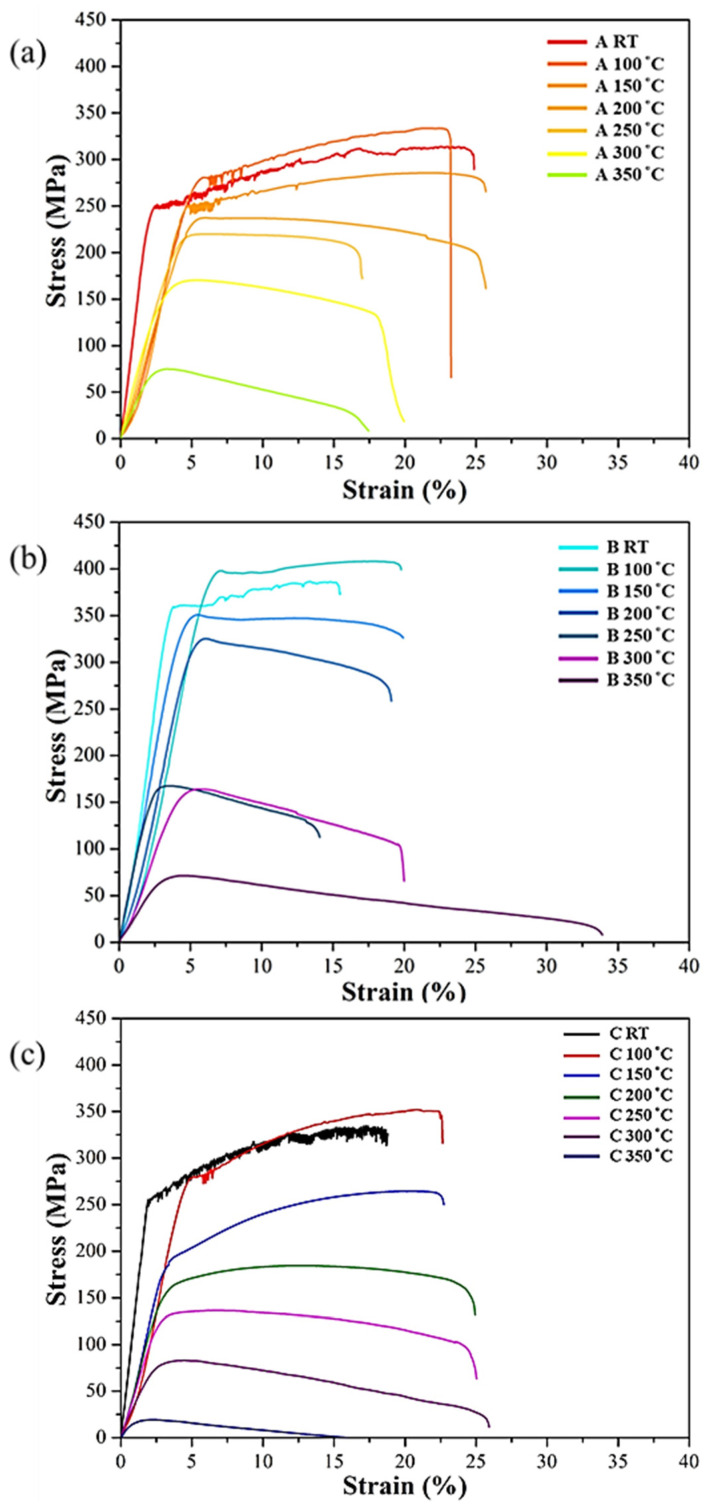
High-temperature tensile stress–strain curve of the SLM Al–Mg–Sc–Zr alloys: (**a**) as-printed and (**b**) single-stage- and (**c**) two-stage-heat-treated specimens.

The high-temperature tensile stress–strain curves at temperatures ranging from room temperature to 350 °C are shown in Figure 6 (data lists in Table 5). All specimens exhibited the highest strength while at 100 °C, decreasing beyond 100 °C. According to the literature [32,33], DSA often induces the characteristics of the Lüders band of uneven plastic deformation. This feature caused internal crack connection, resulting in decreased strength. After DSA was eliminated at 100 °C, SLM Al–Mg–Sc–Zr alloy had the best strength performance. In addition, the serrated jitter in stress–strain curves smoothened owing to sufficient kinetic energy for the dislocation movement and diffusion of solute atoms served by high temperature, which has a tendency toward the plastic deformation mechanism [20,34].

**Figure 7:**
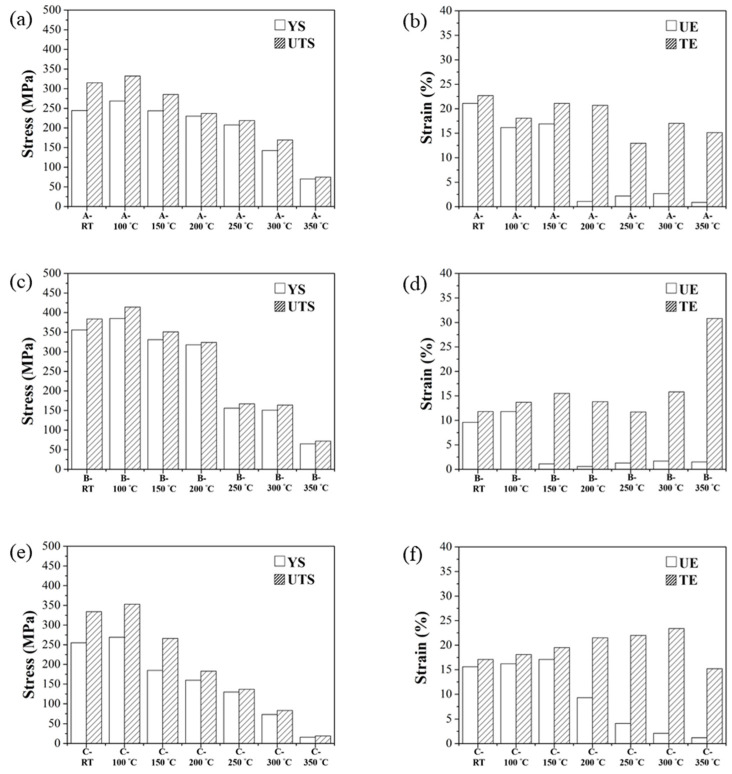
Hightemperature tensile properties of as-printed specimens’ (**a**) strength and (**b**) ductility, single-stage-heat-treated specimens’ (**c**) strength and (**d**) ductility, and two-stage-heat-treated specimens’ (**e**) strength and (**f**) ductility.

Figure 7 shows the high-temperature tensile mechanical properties of three specimens. The single-stage-heat-treated specimens exhibited the highest strength in the range of 100–350 °C [34,35]; however, a dramatic decrease in strength at 250 °C was observed. In addition, the as-printed specimens also revealed a brittle effect (low ductility) at 250 °C. Despite the two-stage-heat-treated specimens’ increasing elongation after 250 °C, their strength is insufficient for industrial applications; therefore, it could be confirmed that the upper limit of the high-temperature applicability of SLM Al–Mg–Sc–Zr alloys is approximately 200 °C [36,37].

The SLM Al alloys were proven to possess significant melting pool texture effects in the previous study, so it is inadequate to evaluate their material properties merely by the tensile results [21]. Hence, the rotation fatigue test was performed in order to further clarify the relationship between texture effect and failure mechanism.

### 3.4. Rotation Fatigue Characteristics

**Figure 8:**
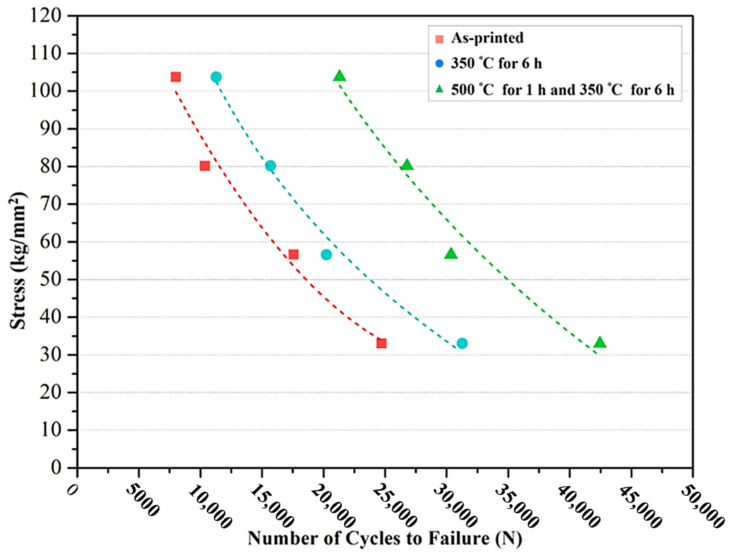
S–N curve of the SLM Al–Mg–Sc–Zr alloys.

**Figure 9:**
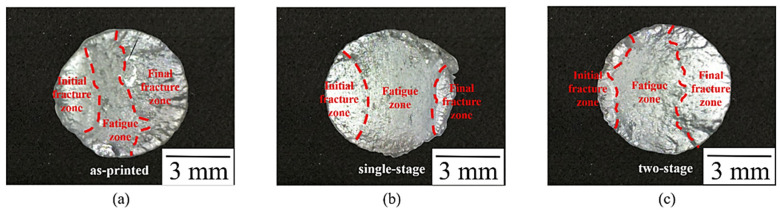
Macrostructure of fatigue fracture of the SLM Al–Mg–Sc–Zr alloys under 7 kg load: (**a**) as-printed and (**b**) single-stage- and (**c**) two-stage-heat-treated specimens.

**Figure 10:**
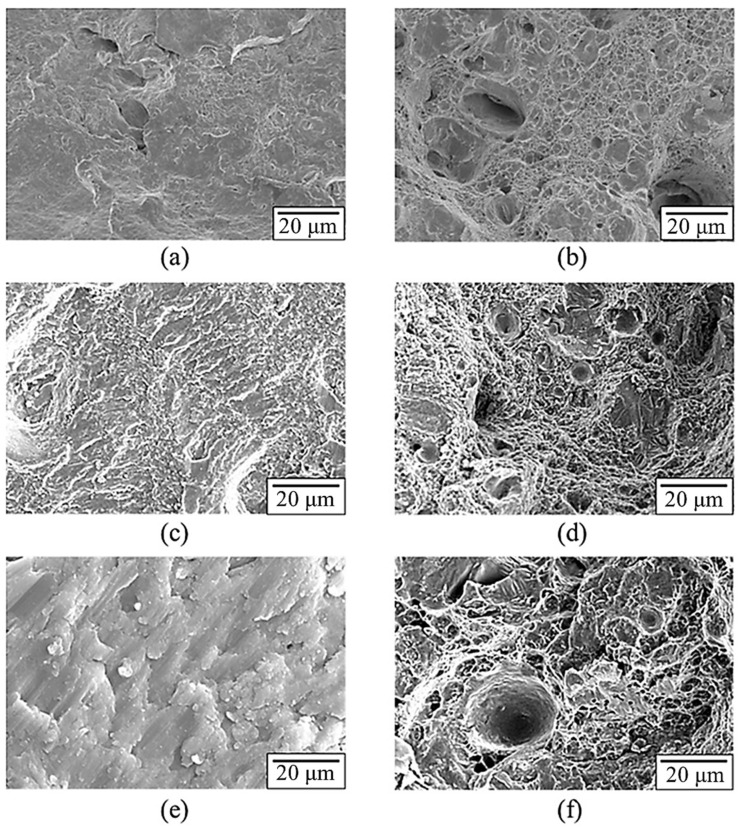
Microstructure of fatigue fracture of the SLM Al–Mg–Sc–Zr alloys under 7 kg load propagation region: (**a**) as-printed and (**c**) single-stage- and (**e**) two-stage-heat-treated specimens; final fracture region: (**b**) as-printed and (**d**) single-stage- and (**f**) two-stage-heat-treated specimens.

Figure 8 presents the rotation fatigue S–N curves (data lists in Table 6). The two-stage heat treatment specimen shows more promising fatigue resistance than the other two specimens. On the basis of macro fatigue fracture morphology (Figure 9), the fatigue-failure section is apparently divided into three zones: initial fracture zone, crack transmission zone, and final fracture zone [38,39]. The three zones of the as-printed specimens are similar in area, while a number of cracks are found on the surface morphology, suggesting brittle fracture characteristics. Compared to the other specimens, the single-stage-heat-treated specimen exhibited the smallest final fracture zone in area; on the other hand, the two-stage-heat-treated specimen showed unapparent brittle fracture characteristics and the largest final fracture zone in area. Figure 10 displays the microscopic morphology of the fatigue fracture surface, with all exhibiting uneven and dimple-like fractures. According to these characteristics, the fatigue mechanism can be inferred as a ductile fracture mechanism [40,41].

### 3.5. Thermal Diffusion and Strengthening Effect during Two-Stage Heat Treatment

**Figure 11:**
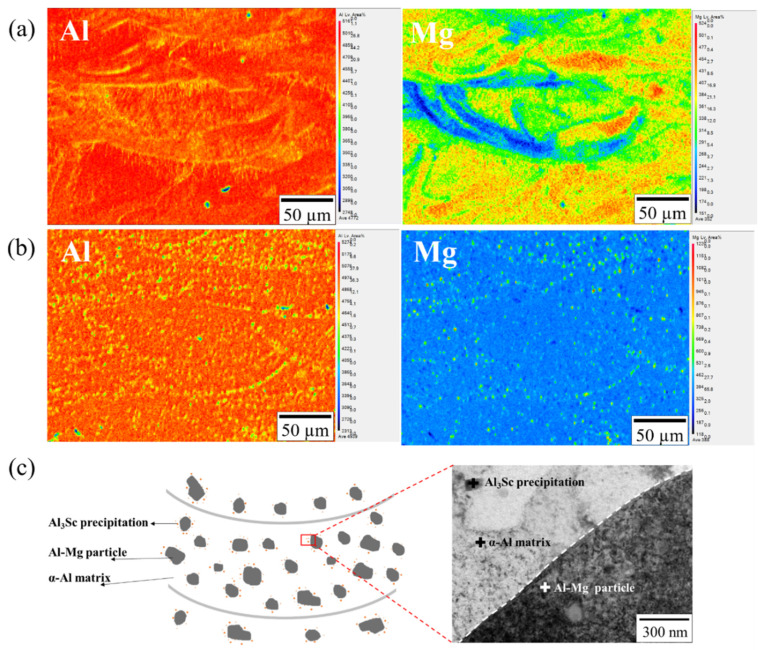
(**a**) EPMA image (Al and Mg elements) of the as-printed specimen; (**b**) EPMA image (Al and Mg elements) of the two-stage-heat-treated specimen; (**c**) schematic diagram and TEM image of the two-stage-heat-treated specimens.

Figure 11a,b demonstrate the EPMA results of as-printed and two-stage-heat-treated specimens, respectively. As observed from the element mapping, the two-stage-heat-treated specimen has an influence on the melting pool structure decomposition and Al–Mg particle formation. In the as-printed melting pool structure, Mg element presents an ununiformly arc-shaped distribution, and the distribution transforms into Mg-rich fine particles homogeneously scattered in Al matrix after two-stage heat treatment. This phenomenon contributes to the improvement of fatigue resistance of the two-stage-heat-treated specimens. Figure 11c offers a schematic presenting the two-stage-heat-treated specimens’ microstructure, with α-Al matrix, Al–Mg particles and Al3Sc precipitation visibly shown in the bright-field TEM image [14].

**Figure 12:**
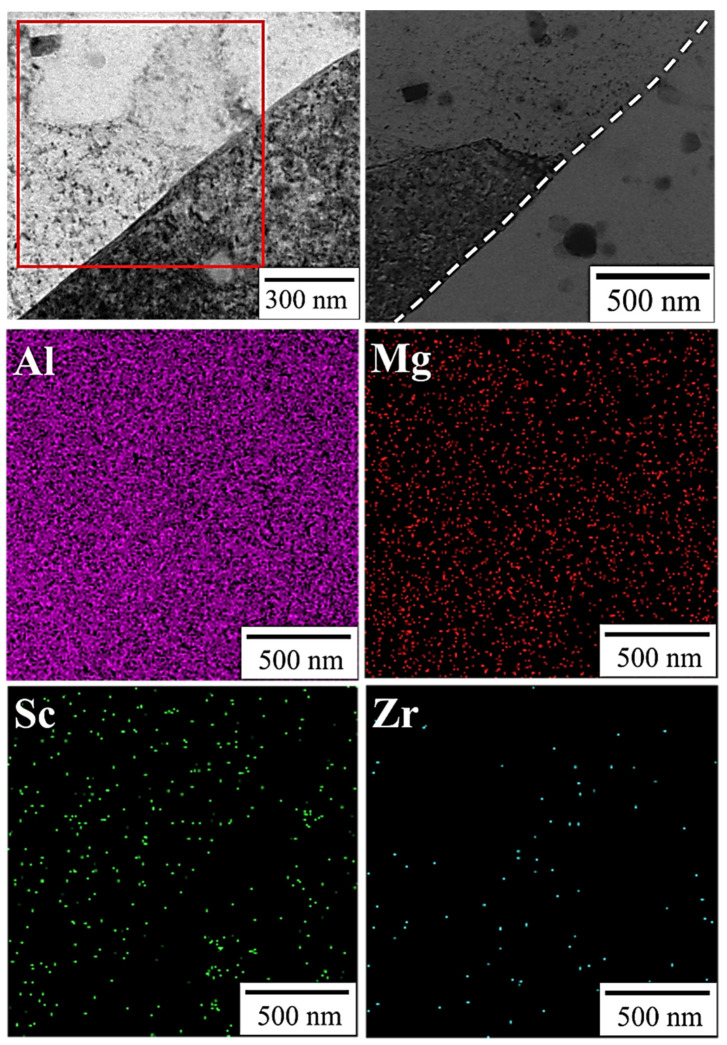
TEM image and EDS mapping of two-stage-heat-treated specimen.

**Figure 13:**
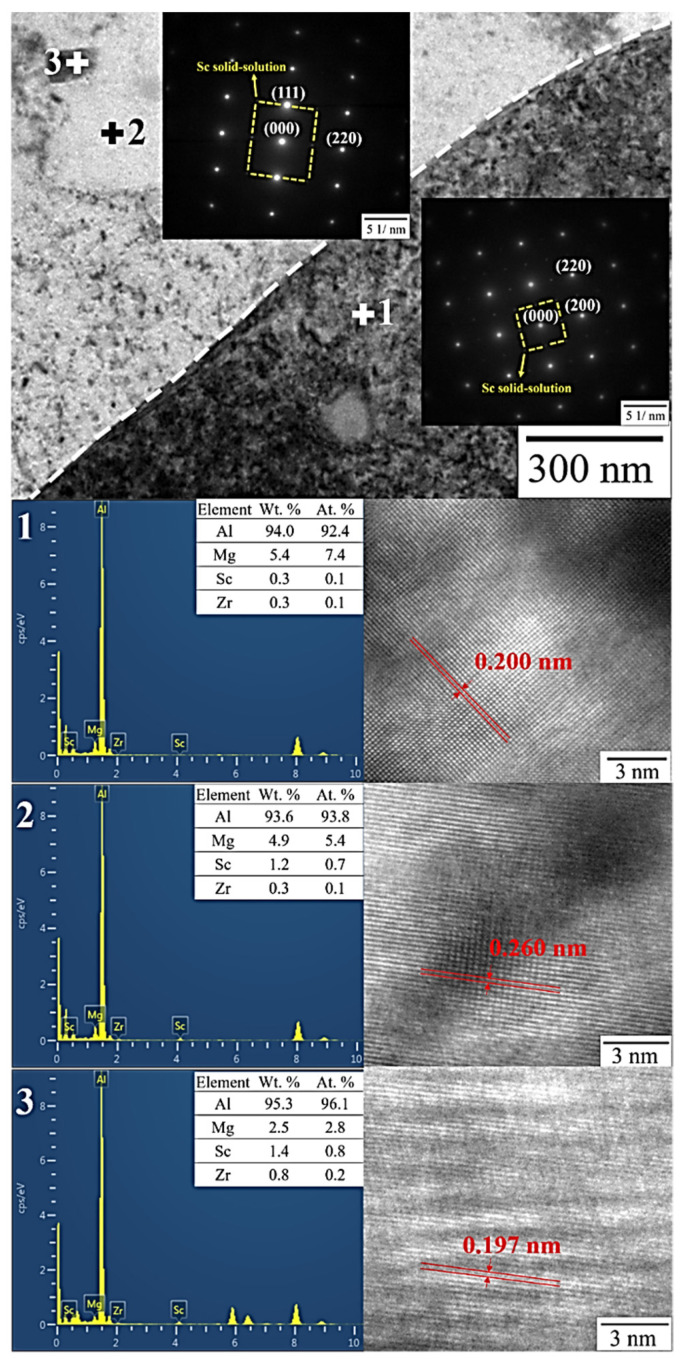
TEM data of two-stage-heat-treated matrix (point (**1**): Al–Mg particle; point (**2**): α-Al matrix; point (**3**): Al_3_Sc precipitation phase).

**Figure 14:**
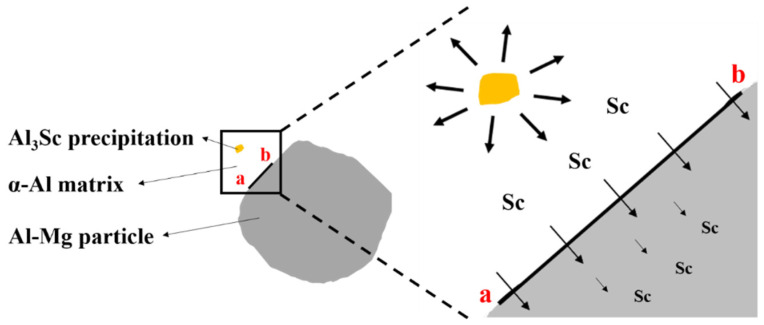
Schematic diagram of atomic diffusion in two-stage-heat-treated SLM Al–Mg–Sc–Zr alloys.

Figure 12 provides the results of TEM equipped with EDS mapping microanalysis on the interface between α-Al matrix and Al–Mg particles, confirming that Mg had homogeneous distribution, but Sc was distributed inside the grains and Zr was distributed in the grain boundary. According to the results of the EDS point analysis and SAED pattern in Figure 13, Al_3_Sc precipitation exhibits the highest Sc content, followed by α-Al matrix, and Al–Mg particles are the least. The most notable point is the observation of Sc solid dissolved in the Al-matrix diffraction pattern (yellow circle in Figure 13 SAED pattern). It is certain that Sc atoms diffused from Al_3_Sc precipitation under two-stage heat treatment contribute to crystal lattice deformation of the α-Al matrix, resulting in a solid solution strengthening effect (schematic as shown in Figure 14) [42,43]. After thermal diffusion, the concentration of Sc has the tendency to achieve equilibrium, and Al_3_Sc precipitation shrink [43,44].

## 4. Conclusions

1. After single-stage heat treatment, Al_3_(Sc, Zr) precipitated at the boundaries with a residual melting pool texture effect increasing its strength but decreasing its ductility. The combination of single-stage heat treatment and solid solution treatment (two-stage heat treatment) decomposes the melting pool structure and induces a homogeneous precipitation, thereby apparently increasing the fatigue resistance.
2. As the tensile temperature increased, the DSA effect of each specimen decreased. At 100 °C, where the DSA effect decreased, SLM Al–Mg–Sc–Zr alloy exhibited the highest high-temperature strength, and the upper limit for high-temperature applications was approximately 200 °C.
3. After the two-stage heat treatment process, the melting pool boundaries of SLM Al–Mg–Sc–Zr alloys decomposed and precipitated homogeneously. The Sc strengthening mechanism was composed of Al_3_Sc precipitation and Sc solid solution after thermal diffusion under heat treatment, increasing matrix strength and inhibiting fatigue crack propagation to provide high fatigue resistance.
4. Overall, the two-stage-heat-treated SLM Al–Mg–Sc–Zr alloy shows better mechanical tensile properties and fatigue resistance, providing wide applicability as an additive manufacturing Al alloy.

## Figures and Tables

**Table 1:** Parameters of the SLM process.

Laser Power	Scanning Speed	Beam Size	Hatch Space	Layer Thickness
300 W	700 mm/s	35 μm	100 μm	30 μm

**Table 2:** Al–Mg–Sc–Zr powder composition.

**Element**	**Al**	**Mg**	**Sc**	**Zr**	**Mn**
Wt.%	Bal.	4.50–5.10	0.68–0.88	0.21–0.52	0.30–0.81
**Element**	**Si**	**Fe**	**Ti**	**O**	**H**
Wt.%	≤0.40	≤0.40	≤0.15	≤0.05	≤0.01

**Table 3:** Heat treatment parameter of SLM Al–Mg–Sc–Zr alloys.

Specimen ID	Specimen Type	Heat Treatment
**A**	as-printed	None
**B**	single-stage heat treatment	350 °C for 6 h/air cooling
**C**	two-stage heat treatment	500 °C for 1 h/water quenching + 350 °C for 6 h/air cooling

**Table 4:** Average tensile properties and hardness of SLM Al–Mg–Sc–Zr alloys.

	YS (MPa)	UTS (MPa)	UE (%)	TE (%)	HRF
**A**	244	315	21.1	22.7	95
**B**	356	384	9.6	11.7	107
**C**	255	334	15.6	17.1	93

**Table 5:** Average high-temperature tensile properties and hardness of SLM Al–Mg–Sc–Zr alloys.

	Temperature	YS (MPa)	UTS (MPa)	UE (%)	TE (%)
**A**	Room temperature	244	315	21.1	22.7
	100 °C	269	332	16.1	18.1
	150 °C	244	285	16.9	21.1
	200 °C	230	237	1.1	20.7
	250 °C	208	219	2.2	13.0
	300 °C	142	169	2.7	17.0
	350 °C	70	75	0.9	15.1
**B**	Room temperature	356	384	9.6	11.8
	100 °C	385	414	11.8	13.7
	150 °C	331	351	1.1	15.5
	200 °C	318	324	0.6	13.8
	250 °C	156	167	1.3	11.7
	300 °C	151	164	1.7	15.8
	350 °C	65	72	1.5	30.8
**C**	Room temperature	255	334	15.6	17.1
	100 °C	268	352	16.1	18.1
	150 °C	185	266	17.1	19.5
	200 °C	160	183	9.6	21.5
	250 °C	130	137	4.1	22.0
	300 °C	73	83	2.1	23.4
	350 °C	16	19	1.2	15.2

**Table 6:** Fatigue resistance of SLM Al–Mg–Sc–Zr alloys.

Load (kg)	Stress (kg/mm^2^)	N (Average Number of Cycles to Failure)
**A (as-printed)**		
7	33.0	24,714
12	56.6	17,571
17	80.2	10,373
22	103.7	8005
**B (single-stage)**		
7	33.0	31,272
12	56.6	20,241
17	80.2	15,696
22	103.7	11,288
**C (two-stage)**		
7	33.0	42,455
12	56.6	30,353
17	80.2	26,785
22	103.7	21,303

## Data Availability

The data presented in this study are available on request from the corresponding author. The data are not publicly available due to privacy or ethics.

## References

[1] Avtokratova E., Sitdikov O., Markushev M., Mulyukov R. (2012). Extraordinary high-strain rate superplasticity of severely deformed Al–Mg–Sc–Zr alloy. Mater. Sci. Eng. A.

[2] Filatov Y.A., Yelagin V.I., Zakharov V.V. (2000). New Al–Mg–Sc alloys. Mater. Sci. Eng. A.

[3] Fang H., Liu H., Yan Y., Luo X., Xu X., Chu X., Lu Y., Yu K., Wang D. (2020). Evolution of Texture, Microstructure, Tensile Strength and Corrosion Properties of Annealed Al-Mg-Sc-Zr Alloys. Mater. Sci. Eng. A.

[4] Barnes A.J., Raman H., Lowerson A., Edwards D. (2013). Recent application of superformed 5083 aluminum alloy in the aerospace industry. Mater. Sci. Forum.

[5] Joy D., Aravindakshan R., Varrma N.S. (2021). Effect of Zirconium additions on microstructure and mechanical properties of hot rolled Al-Mg alloys. Mater. Today Proceedings.

[6] Churyumov A.Y., Pozdniakov A.V., Prosviryakov A.S., Loginova I.S., Daubarayte D.K., Ryabov D.K., Korolev V.A., Solonin A.N., Pavlov M.D., Valchuk S.V. (2019). Microstructure and mechanical properties of a novel selective laser melted Al–Mg alloy with low Sc content. Mater. Res. Express..

[7] Vinogradov A., Washikita A., Kitagawa K., Kopylov V.I. (2003). Fatigue life of fine-grain Al–Mg–Sc alloys produced by equal-channel angular pressing. Mater. Sci. Eng. A.

[8] Sawtell R.R., Jensen C.L. (1990). Mechanical properties and microstructures of Al-Mg-Sc alloys. Metall. Trans. A.

[9] Ma R., Peng C., Cai Z., Wang R., Zhou Z., Li X., Cao X. (2020). Effect of bimodal microstructure on the tensile properties of selective laser melt Al-Mg-Sc-Zr alloy. J. Alloys Compd..

[10] Gu D., Zhang H., Dai D., Ma C., Zhang H., Li Y., Li S. (2020). Anisotropic corrosion behavior of Sc and Zr modified Al-Mg alloy produced by selective laser melting. Corros. Sci..

[11] Kendig K.L., Miracle D.B. (2002). Strengthening mechanisms of an Al-Mg-Sc-Zr alloy. Acta Mater..

[12] Wang Z., Lin X., Kang N., Hu Y., Chen J., Huang W. (2020). Strength-ductility synergy of selective laser melted Al-Mg-Sc-Zr alloy with a heterogeneous grain structure. Addit. Manuf..

[13] Ren L., Gu H., Wang W., Wang S., Li C., Wang Z., Zhai Y., Ma P. (2019). Effect of Mg content on microstructure and properties of Al–Mg alloy produced by the wire arc additive manufacturing method. Materials.

[14] Spierings A.B., Dawson K., Dumitraschkewitz P., Pogatscher S., Wegener K. (2018). Microstructure characterization of SLM-processed Al-Mg-Sc-Zr alloy in the heat treated and HIPed condition. Addit. Manuf..

[15] Sun S., Liu P., Hu J., Hong C., Qiao X., Liu S., Zhang R., Wu C. (2019). Effect of solid solution plus double aging on microstructural characterization of 7075 Al alloys fabricated by selective laser melting (SLM). Opt. Laser Technol..

[16] Chen K.J., Hung F.Y., Lui T.S., Tsai C.L. (2020). Improving the applicability of wear-resistant Al-10Si-0.5Mg alloy obtained through selective laser melting with T6 treatment in high-temperature, and high-wear environments. J. Mater. Res. Technol..

[17] Li R., Wang M., Li Z., Cao P., Yuan T., Zhu H. (2020). Developing a high-strength Al-Mg-Si-Sc-Zr alloy for selective laser melting: Crack-inhibiting and multiple strengthening mechanisms. Acta Mater..

[18] Spierings A.B., Dawson K., Kern K., Palm F., Wegener K. (2017). SLM-processed Sc-and Zr-modified Al-Mg alloy: Mechanical properties and microstructural effects of heat treatment. Mater. Sci. Eng. A.

[19] Kaibyshev R., Musin F., Lesuer D.R., Nieh T.G. (2003). Superplastic behavior of an Al–Mg alloy at elevated temperatures. Mater. Sci. Eng. A.

[20] Musin F., Kaibyshev R., Motohashi Y., Itoh G. (2004). High strain rate superplasticity in a commercial Al–Mg–Sc alloy. Scr. Mater..

[21] Chang K.C., Zhao J.R., Hung F.Y. (2021). Microstructure, Mechanical Properties, and Fatigue Fracture Characteristics of High-Fracture-Resistance Selective Laser Melting Al-Ni-Cu Alloys. Metals.

[22] Besel M., Besel Y., Mercado U.A., Kakiuchi T., Uematsu Y. (2015). Fatigue behavior of friction stir welded Al–Mg–Sc alloy. Int. J. Fatigue.

[23] Li R., Chen H., Chen C., Zhu H., Wang M., Yuan T., Song B. (2019). Selective laser melting of gas atomized Al–3.02 Mg–0.2 Sc–0.1 Zr alloy powder: Microstructure and mechanical properties. Adv. Eng. Mater..

[24] Avtokratova E., Sitdikov O., Mukhametdinova O., Markushev M., Murty S.N., Prasad M.J.N.V., Kashyap B.P. (2016). Microstructural evolution in Al–Mg–Sc–Zr alloy during severe plastic deformation and annealing. J. Alloys Compd..

[25] Rajasekaran S., Udayashankar N.K., Nayak J. (2012). T4 and T6 treatment of 6061 Al-15 vol.% SiCP composite. Int. Math. Res. Not..

[26] Xia S.L., Ma M., Zhang J.X., Wang W.X., Liu W.C. (2014). Effect of heating rate on the microstructure, texture and tensile properties of continuous cast AA 5083 aluminum alloy. Mater. Sci. Eng. A.

[27] Zhang H., Gu D., Dai D., Ma C., Li Y., Cao M., Li S. (2020). Influence of heat treatment on corrosion behavior of rare earth element Sc modified Al-Mg alloy processed by selective laser melting. Appl. Surf. Sci..

[28] Cottrell H., Jaswon M.A. (1949). Distribution of Solute Atoms Round a Slow Dislocation, P. Roy. Soc. Lond. A Mat..

[29] Cottrell A.H. (1953). A note on the Portevin-Le Chatelier Effect. Lond. Edinb. Dublin Philos. Mag. J. Sci..

[30] Shin J.H., Rim G.Y., Kim S.D., Jang J.H., Park S.J., Lee J. (2020). Effects of aging heat-treatment on dynamic strain aging behavior in high-Mn lightweight steel. Mater. Charact..

[31] Zhou P., Song Y., Hua L., Lu J., Zhang J., Wang F. (2019). Mechanical behavior and deformation mechanism of 7075 aluminum alloy under solution induced dynamic strain aging. Mater. Sci. Eng. A.

[32] Robinson J.M., Shaw M.P. (1994). Microstructural and mechanical influences on dynamic strain aging phenomena. Int. Mater. Rev..

[33] Rodriguez P. (1984). Serrated plastic flow. Bull. Mater. Sci..

[34] Guo H., Yan P.F., Wang Y.B., Tan J., Zhang Z.F., Sui M.L., Ma E. (2007). Tensile ductility and necking of metallic glass. Nat. Mater..

[35] Ling Y. (1996). Uniaxial true stress-strain after necking. AMP J. Technol..

[36] Knysh P., Korkolis Y.P. (2017). Identification of the post-necking hardening response of rate-and temperature-dependent metals. Int. J. Solids Struct.

[37] Lin J. (2003). Selection of material models for predicting necking in superplastic forming. Int. J. Plast..

[38] Bahaideen F.B., Saleem A.M., Hussain K., Ripin Z.M., Ahmad Z.A., Samad Z., Badarulzaman N.A. (2009). Fatigue behaviour of aluminum alloy at elevated temperature. Mod. Appl. Sci..

[39] Nakamura Y., Sakai T., Hirano H., Chandran K.R. (2010). Effect of alumite surface treatments on long-life fatigue behavior of a cast aluminum in rotating bending. Int. J. Fatigue.

[40] Kuwamura H. (1997). Transition between fatigue and ductile fracture in steel. J. Struct. Eng..

[41] Farfan S., Rubio-Gonzalez C., Cervantes-Hernandez T., Mesmacque G. (2004). High cycle fatigue, low cycle fatigue and failure modes of a carburized steel. Int. J. Fatigue.

[42] Hiraiwa C., Han D., Kuramitsu A., Kuwabara A., Takeuchi H., Majima M., Uda T. (2013). Chemical expansion and change in lattice constant of Y-doped BaZrO_3_ by hydration/dehydration reaction and final heat-treating temperature. J. Am. Ceram. Soc..

[43] Chang K.C., Zhao J.R., Hung F.Y. (2021). Effects of Hyper-High-Temperature Solid-Solution Treatment on Microstructure Evolution and Nanoprecipitation of the Al-Ni-Cu-Fe-Zr-Sc Alloy Manufactured by Selective Laser Melting. J. Alloys Compd..

[44] Kilian R., Heilbronner R., Stünitz H. (2011). Quartz grain size reduction in a granitoid rock and the transition from dislocation to diffusion creep. J. Struct. Geol..

